# Crystal structure of 7,8-di­chloro-4-oxo-4*H*-chromene-3-carbaldehyde

**DOI:** 10.1107/S205698901501275X

**Published:** 2015-07-08

**Authors:** Yoshinobu Ishikawa

**Affiliations:** a School of Pharmaceutical Sciences, University of Shizuoka, 52-1 Yada, Suruga-ku, Shizuoka 422-8526, Japan

**Keywords:** crystal structure, π–π stacking, hydrogen bond, halogen bond, halogen–halogen inter­action

## Abstract

In the title compound, C_10_H_4_Cl_2_O_3_, a dichlorinated 3-formyl­chromone derivative, the fused-ring system is slightly puckered [dihedral angle between the benzene and pyran rings = 3.66 (10)°]. The dihedral angle between the pyran ring and the formyl plane is 8.64 (7)°. In the crystal, mol­ecules are linked through π–π stacking inter­actions [centroid–centroid distance between the benzene and pyran rings = 3.727 (2) Å], C—H⋯O hydrogen bonds and short C⋯O contacts [2.838 (4) Å]. Halogen bonds between the formyl O atoms and the Cl atoms at the 7-position [Cl⋯O = 2.984 (3) Å, C—Cl⋯O = 170.83 (12)° and Cl⋯O—C = 116.05 (19)°] are also formed along the *a* axis, resulting in helical structures constructed by C—H⋯O hydrogen bonds and Cl⋯O halogen bonds along the *b* axis. In addition, type II halogen–halogen contacts between the chlorine atoms at the 7- and 8-positions [Cl⋯Cl = 3.519 (2) Å, C–Cl⋯Cl = 171.24 (10)° and 88.74 (11)°] are observed.

## Abstract 2

In the crystal of this dichlorinated 3-formyl­chromone derivative, mol­ecules are linked through stacking inter­actions, C—H⋯O hydrogen bonds and short C⋯O contacts. Halogen bonds between the formyl O and Cl atoms and type II halogen–halogen contacts between the Cl atoms are also formed.

## Chemical context   

Halogen bonding and halogen–halogen inter­actions have recently attracted much attention in medicinal chemistry, chemical biology, supra­molecular chemistry and crystal engin­eering (Auffinger *et al.*, 2004 ▸; Metrangolo *et al.*, 2005 ▸; Wilcken *et al.*, 2013 ▸; Sirimulla *et al.*, 2013 ▸; Persch *et al.*, 2015 ▸). Halogen bonding is defined as a net attractive inter­action between an electrophilic region of a halogen atom in a mol­ecule and a nucleophilic region of an atom in a mol­ecule, and is characterized by a short contact between the two atoms. Halogen–halogen inter­actions are generally classified into two categories, type I (*θ*_1_ = *θ*_2_) and type II (*θ*_1_ = 180, *θ*_2_ = 90) where *θ*_1_ and *θ*_2_ are the two C—Cl⋯Cl angles. The type I contact is considered to be van der Waals, and the type II is halogen bonding, *i.e.*, an electrostatic inter­action (Mukherjee *et al.*, 2014 ▸; Metrangolo *et al.*, 2014 ▸).
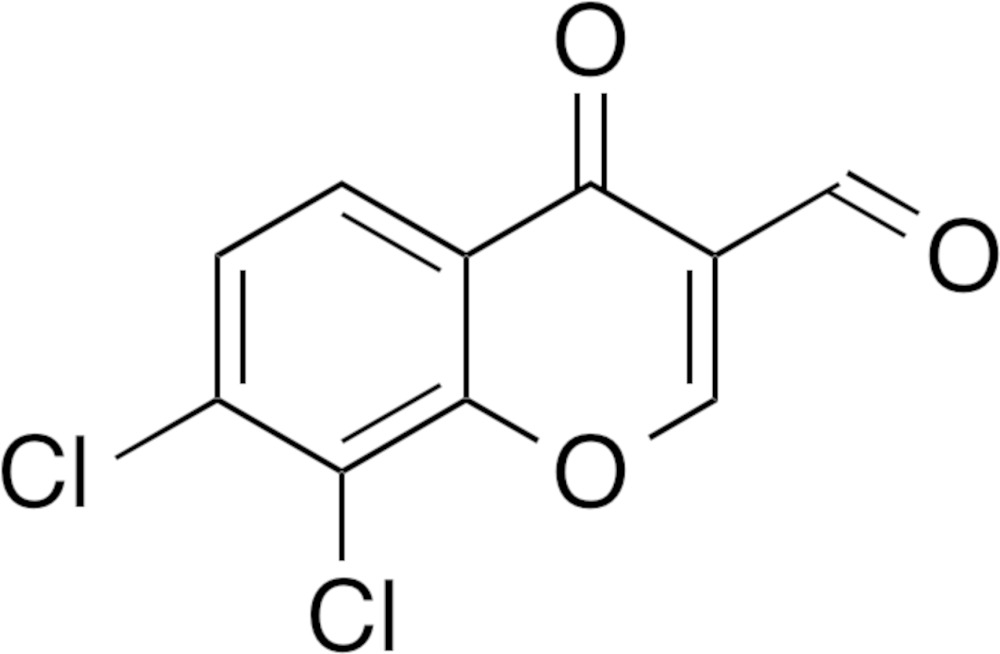


**Figure 1:**
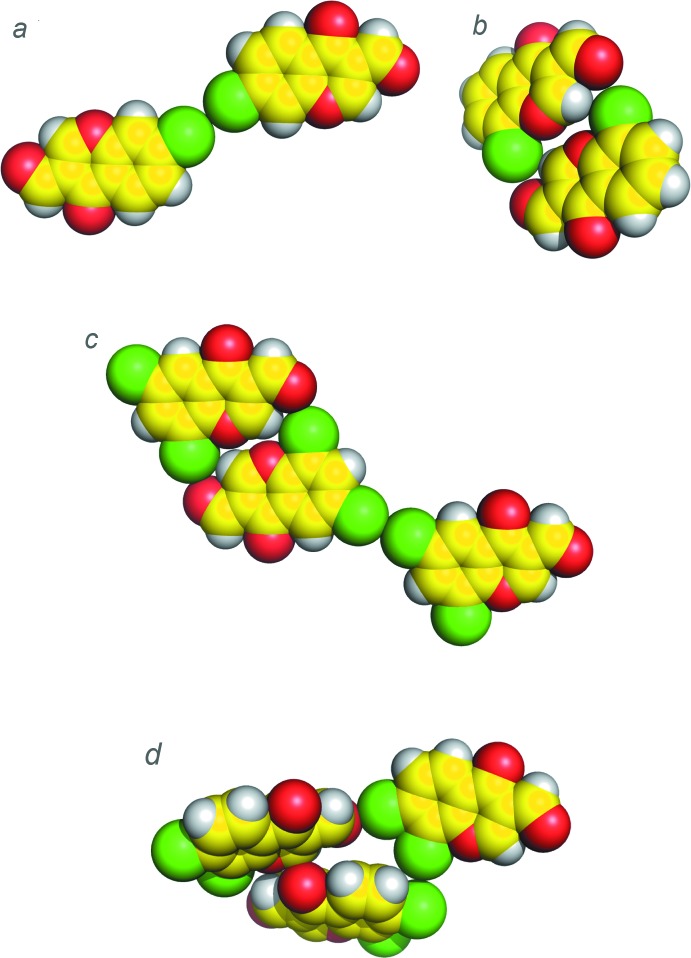
Sphere models of the crystal structures of (*a*) 7-chloro-4-oxo-4*H*-chromene-3-carbaldehyde (Ishikawa, 2014*b* ▸), (*b*) 8-chloro-4-oxo-4*H*-chromene-3-carbaldehyde (Ishikawa, 2014*a* ▸), (*c*) 6,8-di­chloro-4-oxochromene-3-carbaldehyde (Ishikawa & Motohashi, 2013 ▸) and (*d*) the title compound.

I have recently reported the crystal structures of chlorinated 3-formyl­chromone derivatives 7-chloro-4-oxo-4*H*-chromene-3-carbaldehyde (Ishikawa, 2014*b* ▸), 8-chloro-4-oxo-4*H*-chromene-3-carbaldehyde (Ishikawa, 2014*a* ▸) and 6,8-di­chloro-4-oxochromene-3-carbaldehyde (Ishikawa & Motohashi, 2013 ▸). As for the monochlorinated 3-formyl­chromones, a type I van der Waals contact is observed in 7-chloro-4-oxo-4*H*-chromene-3-carbaldehyde (Fig. 1 ▸ *a*), and a van der Waals contact is observed between the formyl oxygen atom and the chlorine atom in 8-chloro-4-oxo-4*H*-chromene-3-carbaldehyde (Fig. 1 ▸ *b*). On the other hand, as for the dichlorinated 3-formyl­chromone, halogen bonding between the formyl oxygen atom and the chlorine atom at the 8-position and a type I short halogen–halogen contact between the chlorine atoms at the 6-position are observed in 6,8-di­chloro-4-oxochromene-3-carbaldehyde (Fig. 1 ▸ *c*). As part of our investigation into these types of chemical bonding, I herein report the crystal structure of a dichlorinated 3-formyl­chromone, 7,8-di­chloro-4-oxo-4*H*-chromene-3-carbaldehyde. The main objective of this study is to reveal the inter­action modes of the chlorine substituents of the title compound in the solid state.

## Structure commentary   

**Figure 2:**
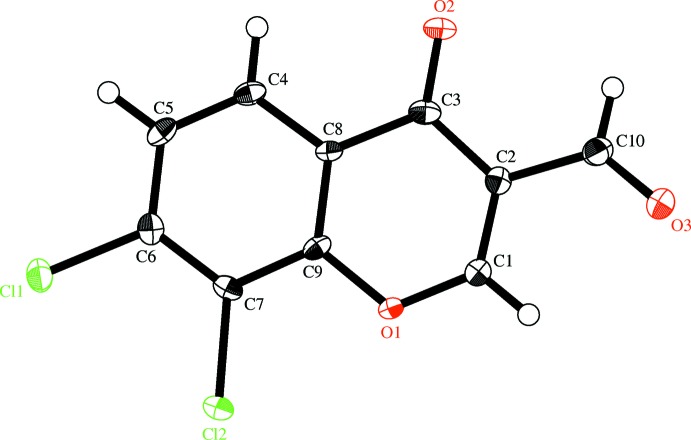
The mol­ecular structure of the title compound, with displacement ellipsoids drawn at the 50% probability level. H atoms are shown as small spheres of arbitrary radius.

The mol­ecular structure of the title compound is shown in Fig. 2 ▸. The fused-ring system is slightly puckered [dihedral angle between the benzene and pyran rings = 3.66 (10)°]. The dihedral angle between the pyran ring and the formyl plane is 8.64 (7)°.

## Supra­molecular features   

**Figure 3:**
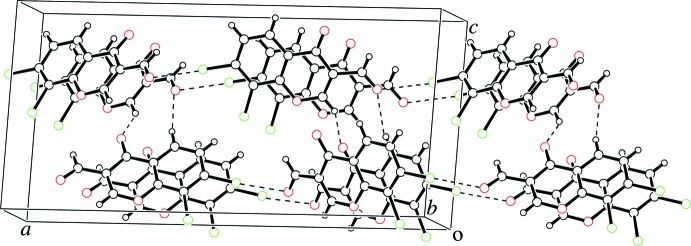
A packing view of the title compound. C—H⋯O hydrogen bonds and Cl⋯O halogen bonds are represented as dashed lines.

In the crystal, the mol­ecules are linked through π–π stacking inter­actions between mol­ecules related by translation-symmetry^i^ [centroid–centroid distance between the benzene and pyran rings of the 4*H*-chromene units = 3.727 (2) Å; symmetry code: (i) *x*, *y* + 1, *z*], and through C—H⋯O hydrogen bonds that involve C1/O2 and C4/O3 atoms, as shown in Fig. 3 ▸ and listed in Table 1 ▸.

Halogen bonds are formed between the chlorine atom at the 7-position and the formyl oxygen atom^ii^ along the *a*-axis direction [Cl1⋯O3^ii^ = 2.984 (3) Å, C6–Cl1⋯O3^ii^ = 170.83 (12)°, Cl1⋯O3^ii^–C10^ii^ = 116.05 (19)°; symmetry code (ii) *x* − 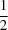, −*y*, *z*], resulting in helical structures constructed by C—H⋯O hydrogen bonds and Cl⋯O halogen bonds along the *b*-axis direction, as shown in Figs. 3 ▸ and 4 ▸. In addition, type II halogen–halogen contacts are observed between the chlorine atoms at the 7- and 8-position^iii^ [Cl1⋯Cl2^iii^ = 3.519 (2) Å, C7^iii^–Cl2^iii^⋯Cl1 = 171.24 (10)°, C6–Cl1⋯Cl2^iii^ = 88.74 (11)°; symmetry code (iii) −*x*, −*y*, *z* + 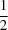], as shown in Fig. 1 ▸ *d*. These electrostatic inter­actions around the chlorine atoms in the title compound are likely due to the cooperativity of the electron-withdrawing chlorine atoms at the 7- and 8-positions. Thus, it is suggested that the chlorine atoms should make their σ-holes larger, and their electropositive regions contact the electronegative regions of the oxygen and chlorine atoms.

In addition to the C—H⋯O hydrogen bonds, halogen bonds and type II halogen–halogen contacts, an unusually short contact is revealed between the α,β-unsaturated carbonyl O2 and the C—H group of C1^iv^ [2.838 (4) Å; symmetry code (iv) –*x* + 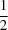, *y*, *z* + 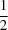; Fig. 1 ▸ *d*]. This inter­esting feature is possibly caused by a dipole–dipole inter­action between the O atom and the C—H group that is enhanced by the polarizing effect of the two chlorine atoms at the 7- and 8-positions of the chromone ring. These observations should be helpful in understanding inter­actions of halogenated ligands with proteins, and thus valuable for rational drug design.

## Synthesis and crystallization   

3′,4′-Di­chloro-2′-hy­droxy­aceto­phenone was prepared from 2,3-di­chloro­phenol by a Fries rearrangement reaction. To a solution of 3′,4′-di­chloro-2′-hy­droxy­aceto­phenone (5.9 mmol) in *N*,*N*-di­methyl­formamide (20 ml) was added dropwise POCl_3_ (11.7 mmol) at 273 K. After the mixture had been stirred for 14 h at room temperature, water (100 ml) was added. The precipitates were collected, washed with water, and dried *in vacuo* (yield: 64%). ^1^H NMR (400 MHz, CDCl_3_): *δ* = 7.82 (*d*, 1H, *J* = 8.8 Hz), 8.08 (*d*, 1H, *J* = 8.8 Hz), 9.05 (*s*, 1H), 10.10 (*s*, 1H). Single crystals suitable for X-ray diffraction were obtained by slow evaporation of a tetra­hydro­furan solution of the title compound at room temperature.

## Refinement   

Crystal data, data collection and structure refinement details are summarized in Table 2 ▸. The C-bound hydrogen atoms were placed in geometrical positions and refined using a riding model [C—H 0.95 Å, *U*_iso_(H) = 1.2*U*_eq_(C)].

## Supplementary Material

Crystal structure: contains datablock(s) General, I. DOI: 10.1107/S205698901501275X/zl2631sup1.cif

Structure factors: contains datablock(s) I. DOI: 10.1107/S205698901501275X/zl2631Isup2.hkl

Supporting information file. DOI: 10.1107/S205698901501275X/zl2631Isup3.cml

CCDC reference: 1410048

Additional supporting information:  crystallographic information; 3D view; checkCIF report

## Figures and Tables

**Figure 4:**
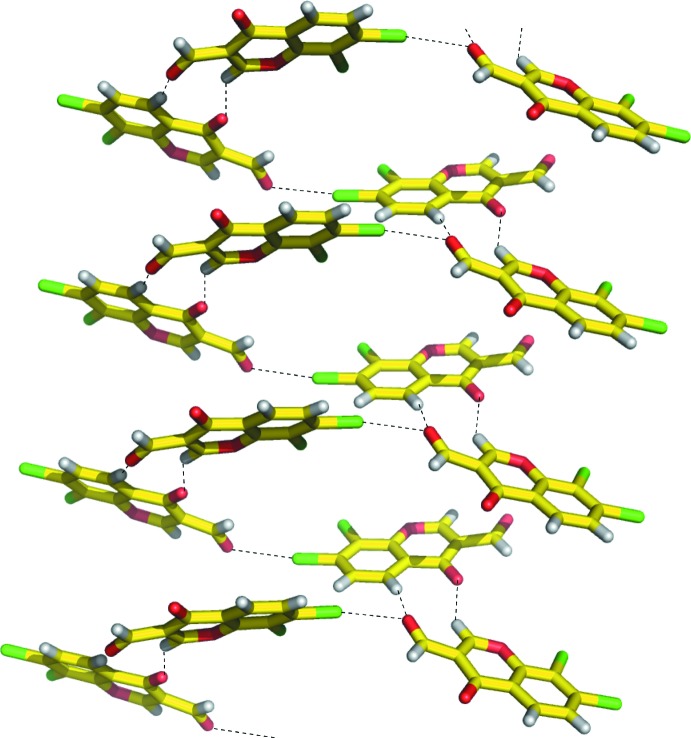
A helical structure constructed by C—H⋯O hydrogen bonds and Cl⋯O halogen bonds in the crystal packing.

**Table 1:** Hydrogen-bond geometry (, )

*D*H*A*	*D*H	H*A*	*D* *A*	*D*H*A*
C1H1O2^i^	0.95	2.28	3.113(4)	146(1)
C4H2O3^ii^	0.95	2.47	3.398(4)	167(1)
C6Cl1O3^iii^	1.72(1)	2.98(1)	4.693(4)	171(1)
C10O3Cl1^iv^	1.21(1)	2.98(1)	3.678(4)	116(1)
C6Cl1Cl2^v^	1.72(1)	3.52(1)	3.884(4)	89(1)
C7Cl2Cl1^vi^	1.72(1)	3.52(1)	5.229(4)	171(1)

Symmetry codes: (i) 
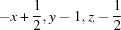
; (ii) 
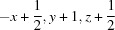
; (iii) 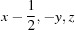; (iv) 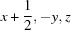; (v) 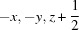; (vi) 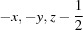.

**Table 2:** Experimental details

Crystal data	
Chemical formula	C_10_H_4_Cl_2_O_3_
*M*_r_	243.05
Crystal system, space group	Orthorhombic, *P* *c* *a*2_1_
Temperature (K)	100
*a*, *b*, *c* ()	23.091(7), 3.7704(18), 10.729(5)
*V* (^3^)	934.1(7)
*Z*	4
Radiation type	Mo *K*
(mm^1^)	0.67
Crystal size (mm)	0.41 0.12 0.05

Data collection	
Diffractometer	Rigaku AFC7R
No. of measured, independent and observed [*F* ^2^ > 2.0(*F* ^2^)] reflections	1571, 1291, 1155
*R*_int_	0.015
(sin /)_max_ (^1^)	0.650

Refinement	
*R*[*F* ^2^ > 2(*F* ^2^)], *wR*(*F* ^2^), *S*	0.025, 0.057, 1.05
No. of reflections	1291
No. of parameters	136
No. of restraints	1
H-atom treatment	H-atom parameters constrained
_max_, _min_ (e ^3^)	0.24, 0.27
Absolute structure	Flack (1983 ▸), 169 Friedel pairs
Absolute structure parameter	0.06(8)

Computer programs: *WinAFC Diffractometer Control Software* (Rigaku, 1999 ▸), *SIR2008* (Burla *et al.*, 2007 ▸), *SHELXL97* (Sheldrick, 2008 ▸) and *CrystalStructure* (Rigaku, 2010 ▸).

## supplementary crystallographic information

### Crystal data

**Table:**

C_10_H_4_Cl_2_O_3_	*F*(000) = 488.00
*M**_r_* = 243.05	*D*_x_ = 1.728 Mg m^−^^3^
Orthorhombic, *P**c**a*2_1_	Mo *K*α radiation, λ = 0.71069 Å
Hall symbol: P 2c -2ac	Cell parameters from 22 reflections
*a* = 23.091 (7) Å	θ = 15.8–17.3°
*b* = 3.7704 (18) Å	µ = 0.67 mm^−^^1^
*c* = 10.729 (5) Å	*T* = 100 K
*V* = 934.1 (7) Å^3^	Plate, yellow
*Z* = 4	0.41 × 0.12 × 0.05 mm

### Data collection

**Table:**

Rigaku AFC–7R diffractometer	θ_max_ = 27.5°
ω scans	*h* = 0→29
1571 measured reflections	*k* = −4→2
1291 independent reflections	*l* = −7→13
1155 reflections with *F*^2^ > 2.0σ(*F*^2^)	3 standard reflections every 150 reflections
*R*_int_ = 0.015	intensity decay: 1.1%

### Refinement

**Table:**

Refinement on *F*^2^	Hydrogen site location: inferred from neighbouring sites
*R*[*F*^2^ > 2σ(*F*^2^)] = 0.025	H-atom parameters constrained
*wR*(*F*^2^) = 0.057	*w* = 1/[σ^2^(*F*_o_^2^) + (0.0252*P*)^2^ + 0.2201*P*] where *P* = (*F*_o_^2^ + 2*F*_c_^2^)/3
*S* = 1.05	(Δ/σ)_max_ < 0.001
1291 reflections	Δρ_max_ = 0.24 e Å^−^^3^
136 parameters	Δρ_min_ = −0.27 e Å^−^^3^
1 restraint	Absolute structure: Flack (1983), 169 Friedel pairs
Primary atom site location: structure-invariant direct methods	Absolute structure parameter: 0.06 (8)
Secondary atom site location: difference Fourier map	

### Special details

**Table:**

Refinement. Refinement was performed using all reflections. The weighted *R*-factor (*wR*) and goodness of fit (*S*) are based on *F*^2^. *R*-factor (gt) are based on *F*. The threshold expression of *F*^2^ > 2.0 σ(*F*^2^) is used only for calculating *R*-factor (gt).

### Fractional atomic coordinates and isotropic or equivalent isotropic displacement parameters (Å^2^)

**Table:**

	*x*	*y*	*z*	*U*_iso_*/*U*_eq_	
Cl1	−0.02754 (3)	0.3441 (2)	0.16617 (7)	0.02008 (17)	
Cl2	0.05650 (3)	−0.0003 (3)	−0.03533 (8)	0.01821 (16)	
O1	0.17307 (8)	−0.0690 (6)	0.04845 (18)	0.0137 (5)	
O2	0.24923 (9)	0.3154 (5)	0.36500 (17)	0.0179 (5)	
O3	0.34477 (9)	−0.3040 (6)	0.12579 (19)	0.0254 (6)	
C1	0.22935 (12)	−0.1270 (8)	0.0740 (3)	0.0136 (7)	
C2	0.25652 (12)	−0.0155 (7)	0.1782 (3)	0.0140 (6)	
C3	0.22568 (13)	0.1862 (9)	0.2724 (3)	0.0149 (7)	
C4	0.12566 (13)	0.3790 (8)	0.3362 (3)	0.0156 (7)	
C5	0.06756 (13)	0.4108 (8)	0.3106 (3)	0.0169 (7)	
C6	0.04536 (12)	0.2967 (8)	0.1961 (3)	0.0156 (7)	
C7	0.08124 (12)	0.1435 (8)	0.1075 (3)	0.0132 (7)	
C8	0.16288 (12)	0.2231 (8)	0.2485 (3)	0.0116 (7)	
C9	0.13996 (12)	0.1023 (8)	0.1362 (3)	0.0127 (7)	
C10	0.31843 (13)	−0.1099 (9)	0.1950 (3)	0.0195 (7)	
H1	0.2515	−0.2554	0.0145	0.0163*	
H2	0.1406	0.4626	0.4133	0.0187*	
H3	0.0423	0.5109	0.3710	0.0203*	
H4	0.3385	−0.0119	0.2642	0.0233*	

### Atomic displacement parameters (Å^2^)

**Table:**

	*U*^11^	*U*^22^	*U*^33^	*U*^12^	*U*^13^	*U*^23^
Cl1	0.0144 (3)	0.0234 (4)	0.0224 (4)	0.0021 (3)	0.0007 (4)	0.0024 (4)
Cl2	0.0181 (3)	0.0216 (4)	0.0149 (3)	−0.0005 (4)	−0.0043 (4)	−0.0019 (4)
O1	0.0147 (10)	0.0162 (12)	0.0102 (10)	0.0015 (9)	−0.0001 (9)	−0.0031 (9)
O2	0.0218 (10)	0.0199 (11)	0.0122 (11)	−0.0014 (10)	−0.0039 (10)	−0.0028 (11)
O3	0.0199 (12)	0.0330 (14)	0.0233 (13)	0.0064 (11)	−0.0001 (9)	−0.0089 (12)
C1	0.0154 (13)	0.0113 (16)	0.0140 (15)	0.0000 (12)	0.0013 (13)	0.0014 (13)
C2	0.0152 (14)	0.0118 (14)	0.0148 (15)	0.0014 (11)	0.0005 (13)	0.0027 (16)
C3	0.0214 (15)	0.0128 (16)	0.0106 (14)	−0.0016 (13)	−0.0019 (13)	0.0040 (14)
C4	0.0224 (15)	0.0113 (16)	0.0130 (16)	−0.0017 (13)	0.0003 (13)	−0.0023 (13)
C5	0.0209 (15)	0.0161 (18)	0.0137 (15)	−0.0012 (14)	0.0064 (13)	−0.0008 (13)
C6	0.0148 (13)	0.0111 (15)	0.0208 (18)	0.0000 (12)	0.0011 (12)	0.0024 (14)
C7	0.0152 (14)	0.0106 (15)	0.0139 (15)	−0.0045 (13)	−0.0033 (12)	0.0026 (13)
C8	0.0149 (14)	0.0112 (16)	0.0087 (14)	−0.0025 (12)	−0.0002 (12)	−0.0002 (12)
C9	0.0193 (14)	0.0093 (14)	0.0095 (14)	0.0016 (12)	0.0030 (12)	0.0016 (12)
C10	0.0209 (15)	0.0208 (17)	0.0166 (16)	0.0003 (14)	0.0012 (13)	−0.0017 (14)

### Geometric parameters (Å, º)

**Table:**

Cl1—C6	1.723 (3)	C4—C5	1.375 (5)
Cl2—C7	1.723 (3)	C4—C8	1.403 (4)
O1—C1	1.346 (4)	C5—C6	1.399 (5)
O1—C9	1.374 (4)	C6—C7	1.387 (4)
O2—C3	1.233 (4)	C7—C9	1.399 (4)
O3—C10	1.207 (4)	C8—C9	1.393 (4)
C1—C2	1.349 (5)	C1—H1	0.950
C2—C3	1.452 (4)	C4—H2	0.950
C2—C10	1.484 (5)	C5—H3	0.950
C3—C8	1.479 (4)	C10—H4	0.950
			
Cl1···Cl2	3.1821 (13)	Cl1···H3	2.7981
Cl2···O1	2.850 (3)	O2···H2	2.6203
O1···C3	2.860 (4)	O2···H4	2.6344
O2···C1	3.570 (4)	O3···H1	2.4697
O2···C4	2.880 (4)	C1···H4	3.2719
O2···C10	2.907 (4)	C3···H1	3.2841
O3···C1	2.803 (4)	C3···H2	2.6882
C1···C7	3.587 (4)	C3···H4	2.7113
C1···C8	2.758 (5)	C6···H2	3.2648
C2···C9	2.765 (4)	C7···H3	3.2742
C4···C7	2.803 (4)	C8···H3	3.2642
C5···C9	2.766 (4)	C9···H1	3.1865
C6···C8	2.785 (4)	C9···H2	3.2686
Cl1···Cl2^i^	3.5191 (17)	C10···H1	2.5378
Cl1···O3^ii^	2.984 (3)	H1···H4	3.4723
Cl2···Cl1^iii^	3.5191 (17)	H2···H3	2.3217
O1···O2^iv^	3.533 (3)	Cl1···H3^xi^	3.2315
O1···O2^v^	3.032 (3)	Cl1···H4^ii^	3.4992
O1···C8^vi^	3.434 (4)	Cl2···H3^iii^	3.1499
O1···C9^vi^	3.352 (4)	Cl2···H3^xi^	3.1018
O2···O1^vii^	3.032 (3)	Cl2···H4^v^	3.2415
O2···O1^viii^	3.533 (3)	O1···H1^ix^	3.5807
O2···C1^vii^	2.838 (4)	O1···H4^v^	3.0689
O2···C1^viii^	3.113 (4)	O2···H1^vii^	2.6840
O2···C2^ix^	3.227 (4)	O2···H1^viii^	2.2789
O2···C2^vii^	3.586 (4)	O2···H4^ix^	3.4425
O2···C3^ix^	3.473 (4)	O3···H2^iv^	2.4674
O2···C10^ix^	3.252 (4)	O3···H4^vi^	3.0577
O3···Cl1^x^	2.984 (3)	C1···H1^ix^	3.3865
O3···C2^vi^	3.415 (4)	C2···H1^ix^	3.3630
O3···C4^iv^	3.398 (4)	C3···H1^ix^	3.5280
O3···C10^vi^	3.186 (5)	C3···H1^vii^	3.1298
C1···O2^iv^	3.113 (4)	C3···H1^viii^	3.3849
C1···O2^v^	2.838 (4)	C4···H2^vi^	3.5694
C1···C2^vi^	3.587 (5)	C5···H3^vi^	3.5033
C1···C3^vi^	3.354 (5)	C6···H3^vi^	3.5082
C1···C3^v^	3.597 (5)	C8···H2^vi^	3.4070
C1···C8^vi^	3.445 (5)	C10···H2^iv^	3.5538
C2···O2^vi^	3.227 (4)	C10···H4^vi^	3.5117
C2···O2^v^	3.586 (4)	H1···O1^vi^	3.5807
C2···O3^ix^	3.415 (4)	H1···O2^iv^	2.2789
C2···C1^ix^	3.587 (5)	H1···O2^v^	2.6840
C2···C3^vi^	3.254 (5)	H1···C1^vi^	3.3865
C3···O2^vi^	3.473 (4)	H1···C2^vi^	3.3630
C3···C1^ix^	3.354 (5)	H1···C3^vi^	3.5280
C3···C1^vii^	3.597 (5)	H1···C3^iv^	3.3849
C3···C2^ix^	3.254 (5)	H1···C3^v^	3.1298
C3···C10^ix^	3.510 (5)	H1···H2^iv^	2.9187
C4···O3^viii^	3.398 (4)	H1···H4^v^	3.5172
C4···C8^ix^	3.428 (5)	H2···O3^viii^	2.4674
C4···C9^ix^	3.486 (4)	H2···C4^ix^	3.5694
C5···C6^ix^	3.596 (5)	H2···C8^ix^	3.4070
C5···C7^ix^	3.532 (5)	H2···C10^viii^	3.5538
C6···C5^vi^	3.596 (5)	H2···H1^viii^	2.9187
C6···C7^ix^	3.432 (5)	H3···Cl1^xii^	3.2315
C7···C5^vi^	3.532 (5)	H3···Cl2^i^	3.1499
C7···C6^vi^	3.432 (5)	H3···Cl2^xii^	3.1018
C8···O1^ix^	3.434 (4)	H3···C5^ix^	3.5033
C8···C1^ix^	3.445 (5)	H3···C6^ix^	3.5082
C8···C4^vi^	3.428 (5)	H4···Cl1^x^	3.4992
C8···C9^ix^	3.567 (5)	H4···Cl2^vii^	3.2415
C9···O1^ix^	3.352 (4)	H4···O1^vii^	3.0689
C9···C4^vi^	3.486 (4)	H4···O2^vi^	3.4425
C9···C8^vi^	3.567 (5)	H4···O3^ix^	3.0577
C10···O2^vi^	3.252 (4)	H4···C10^ix^	3.5117
C10···O3^ix^	3.186 (5)	H4···H1^vii^	3.5172
C10···C3^vi^	3.510 (5)		
			
C1—O1—C9	118.3 (3)	C3—C8—C4	121.6 (3)
O1—C1—C2	124.5 (3)	C3—C8—C9	119.5 (3)
C1—C2—C3	120.8 (3)	C4—C8—C9	119.0 (3)
C1—C2—C10	118.3 (3)	O1—C9—C7	116.2 (3)
C3—C2—C10	120.9 (3)	O1—C9—C8	122.3 (3)
O2—C3—C2	123.5 (3)	C7—C9—C8	121.5 (3)
O2—C3—C8	122.4 (3)	O3—C10—C2	123.8 (3)
C2—C3—C8	114.2 (3)	O1—C1—H1	117.736
C5—C4—C8	120.0 (3)	C2—C1—H1	117.719
C4—C5—C6	120.4 (3)	C5—C4—H2	119.983
Cl1—C6—C5	119.3 (3)	C8—C4—H2	119.980
Cl1—C6—C7	120.0 (3)	C4—C5—H3	119.801
C5—C6—C7	120.7 (3)	C6—C5—H3	119.790
Cl2—C7—C6	122.8 (3)	O3—C10—H4	118.100
Cl2—C7—C9	118.8 (2)	C2—C10—H4	118.125
C6—C7—C9	118.3 (3)		
			
C1—O1—C9—C7	−177.5 (3)	C8—C4—C5—C6	−1.6 (5)
C1—O1—C9—C8	1.3 (4)	C8—C4—C5—H3	178.4
C9—O1—C1—C2	−2.9 (4)	H2—C4—C5—C6	178.4
C9—O1—C1—H1	177.1	H2—C4—C5—H3	−1.6
O1—C1—C2—C3	−0.8 (5)	H2—C4—C8—C3	−0.4
O1—C1—C2—C10	178.1 (3)	H2—C4—C8—C9	180.0
H1—C1—C2—C3	179.2	C4—C5—C6—Cl1	−179.4 (3)
H1—C1—C2—C10	−1.9	C4—C5—C6—C7	1.3 (5)
C1—C2—C3—O2	−174.8 (3)	H3—C5—C6—Cl1	0.6
C1—C2—C3—C8	5.5 (4)	H3—C5—C6—C7	−178.7
C1—C2—C10—O3	−6.0 (5)	Cl1—C6—C7—Cl2	1.0 (4)
C1—C2—C10—H4	173.9	Cl1—C6—C7—C9	−178.62 (18)
C3—C2—C10—O3	172.8 (3)	C5—C6—C7—Cl2	−179.7 (3)
C3—C2—C10—H4	−7.2	C5—C6—C7—C9	0.7 (5)
C10—C2—C3—O2	6.3 (5)	Cl2—C7—C9—O1	−3.2 (4)
C10—C2—C3—C8	−173.4 (3)	Cl2—C7—C9—C8	178.01 (18)
O2—C3—C8—C4	−6.1 (5)	C6—C7—C9—O1	176.4 (3)
O2—C3—C8—C9	173.5 (3)	C6—C7—C9—C8	−2.4 (4)
C2—C3—C8—C4	173.6 (3)	C3—C8—C9—O1	3.7 (4)
C2—C3—C8—C9	−6.8 (4)	C3—C8—C9—C7	−177.6 (3)
C5—C4—C8—C3	179.6 (3)	C4—C8—C9—O1	−176.7 (3)
C5—C4—C8—C9	0.0 (4)	C4—C8—C9—C7	2.0 (4)

Symmetry codes: (i) −*x*, −*y*, *z*+1/2; (ii) *x*−1/2, −*y*, *z*; (iii) −*x*, −*y*, *z*−1/2; (iv) −*x*+1/2, *y*−1, *z*−1/2; (v) −*x*+1/2, *y*, *z*−1/2; (vi) *x*, *y*−1, *z*; (vii) −*x*+1/2, *y*, *z*+1/2; (viii) −*x*+1/2, *y*+1, *z*+1/2; (ix) *x*, *y*+1, *z*; (x) *x*+1/2, −*y*, *z*; (xi) −*x*, −*y*+1, *z*−1/2; (xii) −*x*, −*y*+1, *z*+1/2.

### Hydrogen-bond geometry (Å, º)

**Table:**

*D*—H···*A*	*D*—H	H···*A*	*D*···*A*	*D*—H···*A*
C1—H1···O2^iv^	0.95	2.28	3.113 (4)	146 (1)
C4—H2···O3^viii^	0.95	2.47	3.398 (4)	167 (1)
C6—Cl1···O3^ii^	1.72 (1)	2.98 (1)	4.693 (4)	171 (1)
C10—O3···Cl1^x^	1.21 (1)	2.98 (1)	3.678 (4)	116 (1)
C6—Cl1···Cl2^i^	1.72 (1)	3.52 (1)	3.884 (4)	89 (1)
C7—Cl2···Cl1^iii^	1.72 (1)	3.52 (1)	5.229 (4)	171 (1)

Symmetry codes: (i) −*x*, −*y*, *z*+1/2; (ii) *x*−1/2, −*y*, *z*; (iii) −*x*, −*y*, *z*−1/2; (iv) −*x*+1/2, *y*−1, *z*−1/2; (viii) −*x*+1/2, *y*+1, *z*+1/2; (x) *x*+1/2, −*y*, *z*.
